# Indocyanine Green-Guided Lateral Pelvic Sentinel Lymph Node Mapping in Low Rectal Cancer: Toward a Selective Alternative to Systematic Sidewall Lymphadenectomy

**DOI:** 10.7759/cureus.110314

**Published:** 2026-06-05

**Authors:** Mihaela C Misca, Sorin V Petrea, Eduard Catrina, Sorin Aldoescu, Iulian Brezean

**Affiliations:** 1 General Surgery, Carol Davila University of Medicine and Pharmacy, Bucharest, ROU; 2 General Surgery, Dr. I. Cantacuzino Clinical Hospital, Bucharest, ROU

**Keywords:** image-guided surgery, indocyanine green, lateral pelvic lymph node dissection, low rectal cancer, near-infrared fluorescence, sentinel lymph node biopsy, total mesorectal excision

## Abstract

The management of advanced low rectal cancer is shaped by an ongoing tension between two surgical philosophies regarding lateral pelvic lymph node (LPLN) disease. Eastern and Western guidelines diverge on whether systematic lateral lymph node dissection (LLND) should accompany total mesorectal excision (TME), reflecting different views of the locoregional behavior of LPLN involvement and different thresholds for accepting the urinary and sexual morbidity that accompanies full sidewall clearance. In this editorial, we argue that intraoperative interrogation of each individual case offers a way to refine, rather than resolve by regional consensus, this geography-driven dichotomy, and that indocyanine green (ICG) near-infrared fluorescence provides a principled means of individualizing the extent of lateral dissection. Peritumoral submucosal injection of ICG enables real-time visualization of lymphatic drainage and intraoperative identification of lateral pelvic sentinel lymph nodes (LPSLNs), which can be biopsied and submitted for frozen section. It is important to distinguish between the two applications of this signal. As an intraoperative visualization adjunct, ICG improves lateral node retrieval, and the supporting comparative evidence is relatively consistent. As a sentinel-based decision tool for safely omitting lateral dissection when the sentinel node is negative, the concept is promising but rests on a smaller and less mature evidence base; it remains investigational and requires validation in larger prospective studies with long-term oncologic and functional outcomes before it can guide the omission of lateral dissection in practice. We outline the operative protocol used in our department, extended from sentinel node mapping for gynecological malignancies, and place it in the context of contemporary systematic, propensity-matched, and prospective sentinel biopsy series. In our view, fluorescence-assisted selective LLND may serve as a pragmatic bridge between existing paradigms, preserving the oncologic intent of Japanese-style lateral clearance while aligning with Western priorities of minimizing unnecessary morbidity through tailored, image-guided surgery.

## Editorial

The management of advanced low rectal cancer is shaped by patterns of locoregional spread and the risk of pelvic recurrence, and this has produced two distinct treatment cultures. Western practice, codified in European Society for Medical Oncology (ESMO) and National Comprehensive Cancer Network (NCCN) guidelines, treats total mesorectal excision (TME) combined with neoadjuvant chemoradiotherapy (NCRT) as the cornerstone for advanced disease and avoids routine lateral lymph node dissection (LLND) [1,2]. Japanese guidelines, by contrast, advocate TME plus systematic LLND for T3/T4 low rectal tumors, generally without NCRT, on the view that lateral pelvic lymph node (LPLN) disease is locoregional and surgically curable [1,2]. This divergence, however, reflects more than geography: it arises from differences in neoadjuvant treatment paradigms, in the interpretation of lateral nodal disease, in available surgical expertise, and in the functional trade-offs each tradition is willing to accept. We do not suggest that intraoperative biology invalidates either guideline framework. Rather, we argue that the ability to interrogate the individual case intraoperatively offers a means to refine these paradigms, individualizing the extent of lateral dissection rather than defaulting to a regional standard.

The clinical case for taking the lateral compartment seriously is hard to dismiss. As reviewed by Kehagias and colleagues, LPLN metastasis is reported in 8.6-21.0% of mid-to-low rectal tumors and is consistently associated with worse survival and higher local recurrence [1]. The Japanese JCOG0212 experience, summarized in the same review, is particularly instructive: more than half of the local recurrences observed after TME for stage II/III disease (56.8%) arose in the LPLN basin, and the trial’s long-term data did not demonstrate non-inferiority of TME alone versus TME plus LLND for stage III disease [1]. Yet Christou et al. emphasize that routine LLND carries a substantial operative cost: longer operating times, greater blood loss, and high rates of urogenital dysfunction (urinary 11-50%, sexual 16-40%). Much of this morbidity is attributable to autonomic and obturator nerve injury during sidewall dissection [2]. Contemporary minimally invasive series do not eliminate this burden. This is the impasse: a procedure with a credible oncologic rationale that many surgeons are unwilling to perform routinely on patients who may well have pathologically negative lateral nodes.

**Figure 1:**
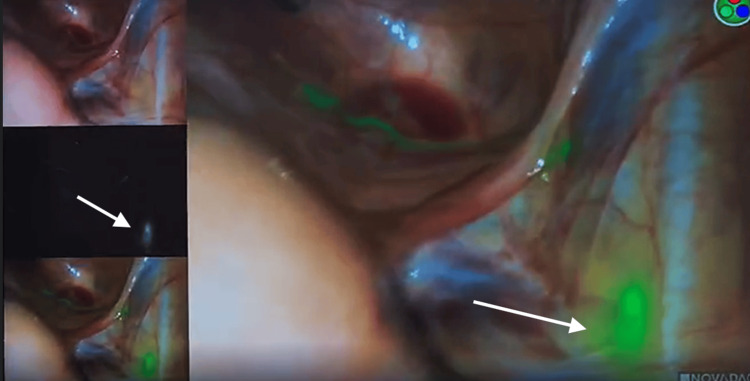
Intraoperative near-infrared fluorescence image of the pelvic sidewall. Following peritumoral submucosal injection of indocyanine green (ICG), a lateral pelvic lymph node is highlighted by fluorescence (arrow). The inset shows the corresponding near-infrared (fluorescence-only) view, in which the node appears as a bright signal against low-background surrounding tissue.

Sentinel lymph node (SLN) mapping rests on the principle that a small number of first-echelon nodes receive initial drainage from the primary tumor and are therefore the earliest sites of metastatic spread. Indocyanine green (ICG) near-infrared fluorescence is well suited to exploit this concept in the pelvis (Figure 1). After peritumoral submucosal injection, the dye’s strong affinity for the lymphatic system produces rapid, distinct fluorescence in lymphatic channels and nodes within minutes; Kehagias et al. observe that the technique offers sufficient tissue penetration and signal-to-background ratio to discriminate lymphatic from non-lymphatic structures, including the autonomic plexus and obturator nerve, in the deep, narrow pelvis [1]. Christou and colleagues describe the same signal serving two complementary uses: it can guide a more thorough conventional LLND, or it can drive a true sentinel strategy in which fluorescent lateral pelvic sentinel lymph nodes (LPSLNs) are biopsied and submitted for frozen section, with the extent of subsequent dissection determined by intraoperative pathology [2]. The latter approach is the one developed in detail by Su et al. for advanced low rectal cancer with lateral nodes present but not enlarged on MRI [3]. These two roles should not be conflated. The evidence that fluorescence improves intraoperative visualization and increases lateral node retrieval derives from comparative cohorts and is relatively robust; the evidence that a negative sentinel node can safely justify omitting full LLND derives chiefly from a single prospective series and pooled diagnostic-accuracy estimates, without long-term oncologic confirmation. The visualization benefit can be adopted with modest risk; the omission decision carries the consequence of leaving potentially involved nodes in situ and therefore demands a higher evidentiary standard before it can be recommended outside trials.

The protocol we propose, which has emerged across other contemporary series, is a submucosal injection on the anal side of the tumor with a fine needle, with a total volume of 4 mL of ICG at 0.1 mg/mL distributed over four peritumoral sites. We extended this indication from our experience with ICG sentinel mapping in gynecological malignancies (incipient cervical neoplasia and endometrial cancer) and adapted it to low rectal cancer while recognizing that the analogy is partial. What transfers is largely technical: the lymphotropic behavior of ICG, near-infrared detection, the operator skill of discriminating fluorescent nodes from autonomic and vascular structures in the deep pelvis, the empirical 10-15-minute uptake window, and the partial overlap of drainage along the internal iliac and obturator territories [4,5]. What does not transfer automatically is the oncologic decision rule: the injection compartment (cervical stroma vs. rectal peritumoral submucosa), tumor biology, and drainage significance differ; most of our gynecological mapping was in treatment-naive disease, whereas rectal mapping often follows NCRT; and sentinel biopsy, a validated staging tool in gynecology, remains investigational as a basis for tailoring lateral dissection in this setting. The injection itself is performed peritumorally in the submucosa immediately after induction of anesthesia. Both Kehagias et al. and Christou et al. describe this regimen as providing reliable lymphatic uptake without the diffusion seen with subserosal administration [1,2]. After induction, laparoscopic TME and high ligation of the inferior mesenteric vessels are completed first, and a near-infrared imaging system is then used to scan the pelvis and define drainage along the internal iliac vessels, the obturator fossa, and the external iliac region. In the sentinel approach described by Su and colleagues, fluorescent LPSLNs are identified at the outset of the lateral phase, sent for frozen section, and the extent of dissection is tailored to the intraoperative pathology [3]. In our department, we propose to perform LPSLN identification and removal immediately at the start of the lateral phase and to submit harvested nodes for frozen section, based on our gynecological experience that pelvic lymphatic drainage occurs rapidly and an SLN can be reliably identified within 10-15 minutes of ICG injection. 

The comparative evidence is consistent. In the propensity-matched cohort reported by Qiu and colleagues, ICG-guided laparoscopic LLND yielded significantly more retrieved lateral lymph nodes, a benefit driven chiefly by the obturator station, with no significant gain in the internal iliac region, together with a shorter postoperative hospital stay and no increase in complications. Intraoperative blood loss and operative time did not differ significantly between the guided and non-guided groups [4]. The consistent benefit across comparative work is thus improved nodal retrieval, concentrated in the more accessible obturator nodes, while operative-quality metrics such as time and blood loss are best described as non-inferior rather than improved. In the Qiu et al. matched analysis, the technical success rate reached 98.3%, with the rare failure attributable to high BMI and thick mesenteric fat [4]. The pooled analysis by Kehagias and colleagues reports a detection rate of 80.7% for lateral nodes and 84.7% for LPSLNs, with their meta-analysis of 248 patients showing 73.7% sensitivity and 100% specificity for sentinel-node detection [1]. The most consequential data, however, come from the prospective sentinel-biopsy series of Su et al.: in advanced low rectal cancers with lateral nodes present but not enlarged on MRI, LPSLNs were identified in 76.2% of patients (mean 2.3 SLNs per case), with complete concordance between frozen section and paraffin histology. When the sentinel nodes were negative, every non-sentinel lateral node was also negative, yielding a false-negative rate of 0 and a negative predictive value of 100% in this cohort [3]. This result is encouraging but must be read as hypothesis-generating rather than definitive. It derives from a single-center series of 23 patients, of whom only 18 had negative sentinel nodes, and without long-term lateral recurrence or functional follow-up, with no false negatives among 18 sentinel-negative patients, the upper bound of the 95% confidence interval for the false-negative rate still approaches 17-18% (rule of three). A point estimate of zero in a small selected cohort, therefore, cannot be equated with the safe omission of lateral dissection. The principle these data suggest is that a negative LPSLN may permit a patient to be spared full LLND, while a positive sentinel node justifies extended dissection, requires validation in larger multicenter prospective studies with long-term endpoints for lateral recurrence and urogenital function before it can be adopted in routine practice [3].

A selective strategy is only as good as its preoperative selection. Both Christou et al. and the Qiu et al. propensity-matched protocols use MRI to define the at-risk population: a short-axis threshold of ≥7 mm is the primary criterion, supplemented for smaller nodes by malignant imaging features, heterogeneous or intense enhancement, irregular shape, rough edges, or high-risk clinicopathologic factors [2,4]. The same criteria have been used in published comparative cohorts to ensure that ICG and non-ICG groups carried equivalent preoperative suspicion of lateral disease. A sentinel-directed strategy is most informative precisely where the Eastern and Western paradigms diverge. Japanese guidelines recommend prophylactic systematic LLND for T3-T4 low rectal tumors below the peritoneal reflection, irrespective of nodal appearance on imaging, whereas Western guidelines rely on neoadjuvant therapy and, in their current form, reserve selective LLND for nodes that are suspicious or persistent on post-treatment MRI. The two traditions now converge at the extreme of frankly suspicious nodes, where both would dissect, and fluorescence serves only to guide a more complete dissection. They diverge as imaging suspicion recedes: for lateral nodes that are visible but not enlarged, the population studied by Su et al., including residual non-enlarged nodes after neoadjuvant therapy, the East would dissect and the West would not; and for patients with no visible lateral nodes, the East would still perform prophylactic dissection while the West would perform none. It is in these zones of divergence that a sentinel-directed approach replaces a geographic rule with a biological one: clear the sidewall only when the sentinel node is positive. In our own practice, the approach is at present reserved for the second of these zones, patients without MRI suspicion of lateral metastasis, who under Japanese guidelines would be candidates for prophylactic LLND, and for whom a negative sentinel node offers a biology-driven alternative aligned with Western restraint. We emphasize that what we describe is a proposed technical approach, extended from our established experience with ICG sentinel mapping in gynecological malignancies, rather than a reported rectal-cancer series; we have not yet accumulated cases of sufficient number or follow-up to report institutional detection rates, frozen-section concordance, or functional outcomes. We note explicitly that, relative to Western practice, this adds a mapping step rather than omitting an otherwise-planned dissection, whereas relative to Japanese practice, it omits prophylactic clearance in sentinel-negative patients. Neoadjuvant therapy must also be accounted for, since radiotherapy may alter lymphatic drainage by blocking nodal pathways. In the Su et al. sentinel-biopsy series, 78.3% of patients had received NCRT and 13.0% had received chemotherapy; only 8.7% went directly to surgery. The same authors observe that fluorescent presentation differs after NCRT, which is why their sentinel protocol specifically targets patients with lateral nodes visible but not enlarged on post-treatment MRI and why they recommend that fluorescence imaging be tightly integrated with the surgical workflow rather than treated as a stand-alone test [3].

Before considering how these observations might be operationalized, we emphasize that the evidence underpinning ICG-guided lateral pelvic sentinel mapping remains preliminary. Kehagias and colleagues characterize the technique explicitly as an emerging procedure, their systematic review resting on single-center retrospective cohorts and small prospective series with relatively short follow-up [1]. Su et al., whose prospective data are the most directly relevant, present lateral pelvic sentinel biopsy as a promising and supplementary tool for predicting lateral nodal status rather than as an established standard; their negative predictive value of 100% derives from only 18 sentinel-negative patients, a point estimate whose confidence interval remains wide [3]. Three further caveats constrain interpretation: the effect of NCRT on lymphatic architecture and fluorescence presentation is incompletely characterized; fluorescence marks lymphatic tissue, not metastatic involvement, so the strategy depends ultimately on accurate pathology; and the retrieval benefit is anatomically uneven, being most reliable in the obturator region and least so for internal iliac nodes lying behind the iliac vessels. For these reasons, we distinguish throughout between the visualization role of fluorescence, for which comparative evidence is relatively consistent, and the sentinel-directed decision to omit lateral dissection, which remains investigational. The algorithm that follows is offered as a framework to structure prospective evaluation, not as a recommendation for current practice.

**Figure 2:**
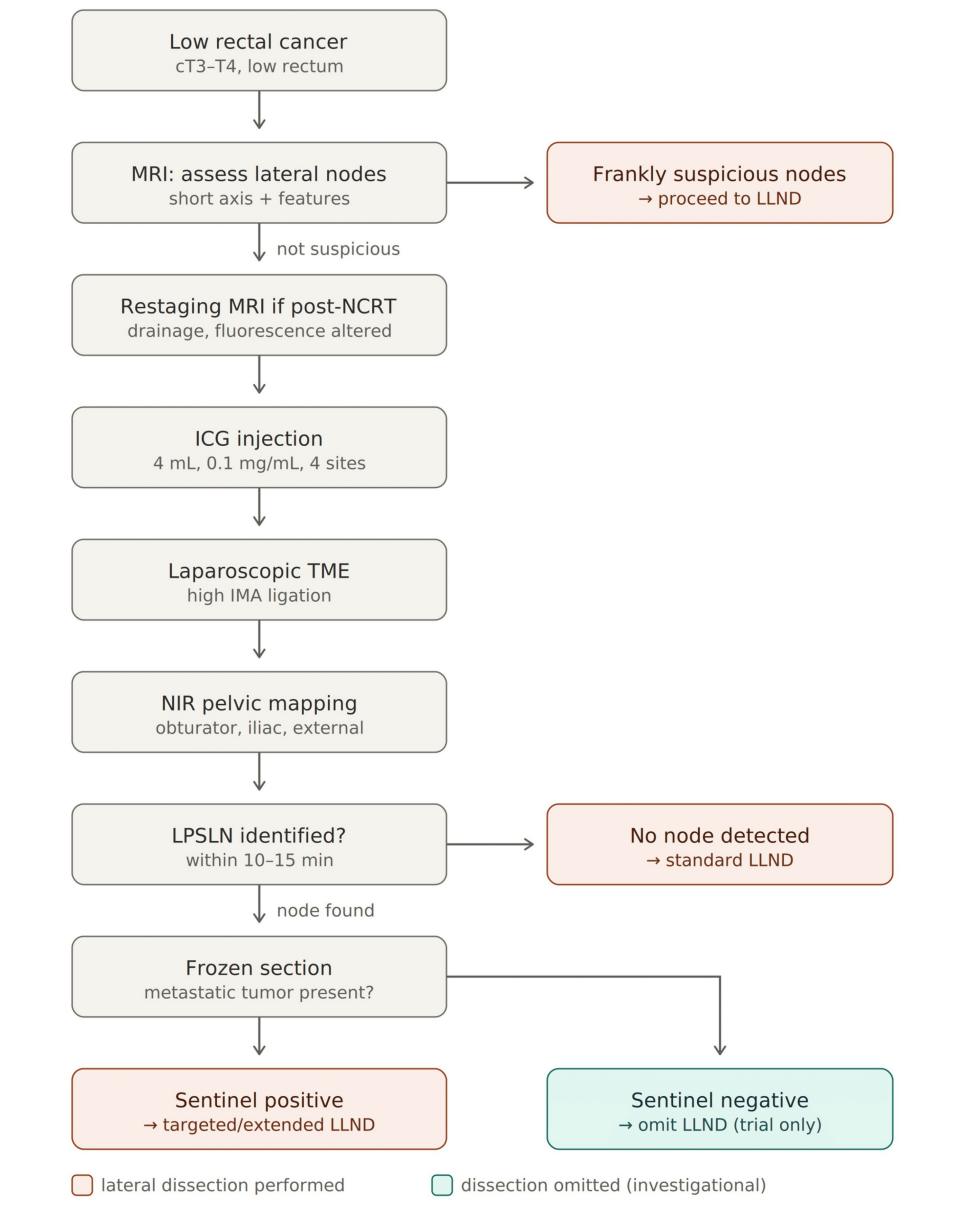
Proposed algorithm for indocyanine green-guided lateral pelvic sentinel lymph node mapping in low rectal cancer. Frankly suspicious nodes proceed directly to lateral lymph node dissection (LLND). In patients with non-suspicious or visible-but-not-enlarged nodes, peritumoral ICG injection is followed by total mesorectal excision and near-infrared mapping; identified sentinel nodes are submitted for frozen section. A positive sentinel node prompts targeted or extended LLND, a non-detected sentinel node defaults to standard LLND, and a negative sentinel node permits omission of LLND within a protocol or trial framework only. NCRT: neoadjuvant chemoradiotherapy; IMA: inferior mesenteric artery; NIR: near-infrared; LPSLN: lateral pelvic sentinel lymph node; ICG: indocyanine green

With these limitations in mind, the same data nonetheless support a provisional, testable algorithm. MRI stratifies patients along the axis where the two paradigms disagree, from frankly suspicious nodes, through nodes visible but not enlarged, to no visible nodes; ICG mapping resolves the individual drainage pattern and identifies the LPSLNs; and intraoperative pathology, not geography, decides the extent of lateral surgery (Figure 2). Patients with positive LPSLNs proceed to targeted or extended LLND, honoring the Japanese principle of thorough locoregional clearance for proven disease. Patients with negative LPSLNs are spared full LLND and its functional cost, in line with Western priorities of avoiding overtreatment (Figure 2).

Stepwise pathway

Preoperative Selection (MRI)

Candidates are patients with low rectal cancer (cT3-T4, below the peritoneal reflection) whose lateral nodes are non-suspicious or visible but not enlarged, short-axis < 7 mm, and lacking malignant features (heterogeneous or intense enhancement, irregular shape, rough edges). Nodes that are frankly suspicious (≥ 7 mm or with malignant features) are not candidates for a sparing decision and proceed to LLND.

Neoadjuvant Therapy and Restaging

In patients receiving NCRT, selection and mapping are based on post-treatment (restaging) MRI, since radiotherapy alters lymphatic drainage and fluorescence presentation; mapping is integrated into the surgical workflow rather than used as a stand-alone test.

ICG Injection

Immediately after induction of anesthesia, submucosal peritumoral injection on the anal side of the tumor with a fine needle, a total of 4 mL of ICG at 0.1 mg/mL distributed over four peritumoral sites.

Surgical Sequence

Complete laparoscopic TME and high ligation of the inferior mesenteric vessels, then scan the pelvis with a near-infrared imaging system to define lateral drainage.

LPSLN Identification

At the start of the lateral phase (within ~10-15 minutes of injection), identify fluorescent LPSLNs across the internal iliac, obturator, and external iliac regions; harvest and submit for frozen section. Detection is most reliable in the obturator region and least reliable for internal iliac nodes lying behind the iliac vessels.

Intraoperative Decision

The extent of lateral dissection is determined by frozen-section pathology (Figure 2).

The geography-driven controversy becomes a biology-driven decision tied to each patient’s own lymphatic anatomy. The functional and economic case follows naturally. The Qiu cohort showed a shorter postoperative hospital stay with no increase in complications, while operative time and blood loss did not differ significantly between groups [4]; in the Su et al. sentinel cohort, complications were infrequent and conservatively managed, and no ICG-related allergic reactions were reported [3]. Reduced morbidity, shorter hospitalization, and avoidance of unnecessary dissection plausibly offset the cost of fluorescence platforms and tracers.

These conclusions must, however, be tempered. As Kehagias and colleagues acknowledge in their own systematic review, the available evidence is dominated by single-center retrospective cohorts and small prospective series with relatively short follow-up; the impact of NCRT on lymphatic architecture and fluorescence patterns is incompletely characterized; and fluorescence marks lymphatic tissue, not metastatic involvement, so the strategy ultimately depends on accurate pathology [1]. Definitive validation will require multicenter prospective trials comparing ICG-guided selective LLND with conventional LLND and with TME plus NCRT alone, using long-term endpoints for local control, survival, and detailed urogenital function. Until such data exist, the selective strategy should be regarded as investigational.

ICG-guided LPSLN mapping, in our view, offers a conceptually coherent and technically feasible synthesis of the Eastern and Western philosophies for low rectal cancer. By centering management on real-time, patient-specific lymphatic mapping rather than on regional practice tradition, it has the potential to deliver the locoregional control of the Japanese approach while preserving the functional outcomes that have driven Western reluctance toward routine LLND. We emphasize, however, that the visualization advantage and the sentinel-directed omission strategy stand on different evidentiary footings: the former is supported by consistent comparative data, whereas the latter remains to be validated. If forthcoming trials confirm the principle established by Su and colleagues, that a negative LPSLN reliably excludes residual lateral disease, and provided that long-term oncologic safety, preservation of urinary and sexual function, reproducibility across centers, and feasibility after neoadjuvant therapy are likewise demonstrated, sentinel-directed selective LLND may become an important component of future individualized treatment strategies for low rectal cancer.
